# Child Injury Prevention: It Is Time to Address the Determinants of Health

**DOI:** 10.3390/children8010046

**Published:** 2021-01-14

**Authors:** Amy E. Peden, Richard C. Franklin

**Affiliations:** 1 School of Population Health, Faculty of Medicine, University of New South Wales, Kensington, NSW 2052, Australia; 2 College of Public Health, Medical and Veterinary Sciences, James Cook University, Townsville, QLD 4811, Australia; richard.franklin@jcu.edu.au

Injuries, although almost entirely preventable, accounted for more than 4.4 million deaths and resulted in over 520 million cases of nonfatal injury-related harm globally in 2017 [1]. Road traffic injuries, falls and drowning are leading injury mechanisms [2,3,4]. However, some population groups are more vulnerable to being injured than others. This Special Issue sought to unpack this concept by exploring the determinants of health and their impact on child injuries.

The determinants of health are a wide group of underlying causes, often referred to as the ”causes of the causes” [5], which impact health and wellbeing. Determinants of health include level of education, family income, housing conditions, and the geographical location of place of residence [6]. While it is clear that determinants of health impact wellbeing and quality of life [7,8,9], more work needs to be done to explore their impact on injury risk. Even more important is the need to identify and quantify the bi-directional benefit of preventing injury and addressing determinants of health [10,11]. This is of importance across all ages; however, the greatest health gains can be made among children and adolescents [12]; hence the focus of this Special Issue. 

There are many examples of how determinants of health impact injury risk. These include geographical location, income level and employment. Geographical location impacts availability of medical services, with rural people often located at a larger distance from emergency services, which impacts response times [13]. There are also persistent healthcare workforce shortages [14] resulting in reduced access to services such as occupational therapy for in-home modifications or physiotherapy to help with strength, balance and posture to prevent falls [15]. 

Similarly, income level impacts injury risk with higher rates of road traffic injuries seen in lower income areas due to differences in roadway design [16]. A low-income household was found to be the single most important predictor for severe paediatric intentional and unintentional injury [17]. Higher rates of fire-related injuries [18] and hot water-related burns and scalds [19] were seen in low-income households, in part due to lower use of protective devices such as smoke alarms and hot water system modifications.

Income level is linked to employment. Research indicates higher rates of suicide [20] and intentional injury [21] among those who are unemployed and higher rates of injury-related fatalities among children in households where no adults are employed [22]. Unemployment is also linked to alcohol and drug abuse [23], another risk factor for a myriad of injury mechanisms. 

Education, another determinant of health, is used to impact injury prevention with schools used to deliver messages and information often provided in written form to parents and caregivers to help inform about child and household safety. For example, programs in Australia, Israel and India use the school system to deliver swimming lessons to reduce drowning risk [24], road safety education [25] and fire safety [26].

This Special Issue comprises ten eclectic papers, spanning high income and low- and middle-income contexts, each of which provides a small window into childhood injuries and highlights the need to consider the determinants of health. Common themes to emerge included the impact of socio-economic status on increased child injury risk across a range of injury mechanisms [27,28,29,30]. The issue of mobile device use leading to distraction from supervision of children at playgrounds is ubiquitous across high, middle, and low socio-economic areas in developed nations, and leads to increased injury risk [31].

Race, ethnicity and culture were common determinants across several papers, with race and ethnicity being identified as risk factors for work-related fatalities in New Zealand [32], oral self-harm among institutionalized children in Romania [33] and among factors increasing drowning risk [29]. Cultural-related barriers were identified as posing a challenge to the implementation of accepted child drowning prevention interventions in the Sundarbans region of India [30]. Similarly, rurality was found to increase child injury risk in Australia [28] and the Sundarbans region [30]. 

More broadly, age- and sex-related factors were identified as impacting injury risk during sports and other physical activities, particularly during adolescence [34,35,36], a period of much physical change. 

We encourage our readers to explore these 10 papers and consider the role that determinants of health play in injury risk in their own context. Determinants of health are important, and it is vital to understand how they impact injury, as addressing them will have the additional benefit of reducing injuries. While it may not always be easy it is clear that more work needs to be undertaken around injury and determinants of health. We must ensure that geography, income level, race and ethnicity and education are not barriers to ensuring safety. 
